# Preeclampsia and Its Complications Exacerbate Development of Postpartum Depression: A Retrospective Cohort Study

**DOI:** 10.1155/2021/6641510

**Published:** 2021-04-22

**Authors:** Ying Ye, Li Chen, Jiani Xu, Qinjin Dai, Xin Luo, Nan Shan, Hongbo Qi

**Affiliations:** ^1^The Department of Obstetrics, The First Affiliated Hospital of Chongqing Medical University, Chongqing 400016, China; ^2^State Key Laboratory of Maternal and Fetal Medicine of Chongqing Municipality, Chongqing Medical University, Chongqing 400016, China; ^3^The Bishan Hospital of Chongqing, Chongqing 402760, China; ^4^Guangzhou Women and Children's Medical Center, Guangzhou Medical University, Guangdong 510623, China

## Abstract

**Background:**

Hypertensive disorders were proved to be associated with the development of depression. But it is unclear if pregnancy-induced hypertensive diseases, especially preeclampsia (PE), will affect postpartum moods. We aimed to determine the incidence rate of postpartum depression (PPD) in PE patients and comprehensively evaluate the association between PPD and PE, including its severity and complications.

**Methods:**

425 participants including 130 PE mothers were enrolled in this retrospective cohort study. Each woman was asked to complete a questionnaire integrating the Edinburgh Postnatal Depression Scale (EPDS), the Leakage Index Questionnaire, and a pain scale questionnaire within 6 weeks after delivery. The EPDS cut-off score above 13 was recognized as screening positive for PPD. Data between groups were compared by bivariate analysis.

**Results:**

PE mothers showed a direct tendency to PPD development. The positive screening for PPD in the PE group was significantly higher than that of the control group (30.77% vs. 14.58%). Based on the results of the regression model, women diagnosed with severe PE and fetal growth restriction were more inclined to develop PPD than normal ones (AOR: 2.759, 95% CI: 1.206-6.315 and AOR: 3.450, 95% CI: 1.596-7.458). It is also indicated that postpartum pain exacerbated the odds of PPD in PE patients (AOR: 1.509, 95% CI: 1.078-2.114).

**Conclusions:**

PE was an independent risk factor for PPD. Its severity and complications exacerbate the development of PPD. Doctors and society should pay more attention to PE patients after delivery against the development of PPD.

## 1. Introduction

Postpartum depression (PPD) is a major depressive episode that begins within 6 weeks after delivery [1]. PPD affects a significant number of mothers, as the global prevalence of PPD reportedly ranges from 3% to 38% [2, 3]. Mothers with PPD often exhibit sadness, loss of interest and joy, feelings of helplessness, difficulty concentrating and remembering, and sleep disturbances. PPD may negatively impact maternal health, parenting, and subsequently the development of children. It may result in abusive parenting, maternal suicide, and infanticide [4, 5]. Besides, it can lead to negative sequelae for the offspring, including delayed cognitive development, behavioral problems, and even suicidal ideation [6–8]. Therefore, identifying the risk factors for PPD is important for earlier detection and prevention of negative consequences of PPD.

This disorder may be caused by multiple risk factors, including the history of depression, preterm delivery, poor marital relationship, and low social income [9, 10]. These factors have been fully elucidated to be associated with PPD, and recently, a few studies have evaluated the effects of pregnant complications on PPD, such as preeclampsia (PE).

As reported, hypertensive disorder in pregnancy (HDP) is a risk factor for depression, and the prevalence is about 20%-30% [11]. PE is one HDP characterized as hypertension developing after 20 weeks of gestation with the coexistence of ≥1 of a new onset of (1) proteinuria, (2) maternal organ dysfunction, or (3) uteroplacental dysfunction [12]. PE is one of the leading causes of maternal/fetal mortality and morbidity worldwide and is responsible for around 60,000 deaths [13]. PE directly threatens mothers and causes various adverse fetal outcomes, leading to small-for-gestational-age babies, premature delivery, and infant death [14].

Our previous study demonstrated that PE patients had nearly 3-fold increased odds for PPD compared to normal women, and patients with severe PE had a more than 4-fold higher risk of screening positive for PPD [15]. However, whether the severity of PE and fetal outcomes would contribute to PPD has not been investigated.

Herein, we aimed to compare the incidence rate of PPD in PE and normal women by employing the EPDS and to comprehensively evaluate the association between PPD and PE, especially its severity and complications. In addition, it has been previously reported that pelvic floor symptoms, urinary incontinence, and pain would affect postpartum moods [16, 17], so we also employed the Leakage Index Questionnaire and the pain scale.

## 2. Methods

### 2.1. Study Design and Participants

In this two-center retrospective cohort study, patients who delivered between October 1, 2018, and August 30, 2019, were enrolled from the First Affiliated Hospital of Chongqing Medical University and Qinghai Red Cross Hospital. All patients were asked to independently complete the questionnaires within about 6 weeks after delivery. With informed consent, the answers of the patients can be used here.

### 2.2. Inclusion and Exclusion Criteria

The inclusion criteria were (1) diagnosis of PE by elevated blood pressure (systolic pressure ≥ 140 mmHg or diastolic pressure ≥ 90 and with proteinuria) after 20 weeks of gestational age according to the ACOG guidelines (2019), (2) diagnosed with PE accompanied with fetal growth restriction (FGR), (3) maternal age ranging from 18 to 45 years, and (4) gestational age ≥ 28 weeks.

The exclusion criteria were as follows: (1) presence of other complications, such as gestational diabetes mellitus, intrahepatic cholestasis of pregnancy, and hyperthyroidism; (2) preterm (gestational age less than 36^+6^ weeks) not caused by PE; (3) preexisting mental diseases, history of depression; and (4) stillbirth or giving birth to a malformed fetus (including any minor anomalies).

### 2.3. Measurements

All details of maternal and neonatal conditions during pregnancy and delivery were obtained from the hospital information systems. After applying the inclusion and exclusion criteria, we invited mothers for a clinical visit within 6 weeks after delivery and encouraged them to participate in our questionnaires, including EPDS, Leakage Index Questionnaire, and pain scale (numerical rating scales).

EPDS is the most commonly used PPD scale worldwide and is one of the most authoritative self-evaluation scales to screen for PPD [18]. Each of its 10 items is divided into 4 grades and scored from 0 to 3. The total score ranges from 0 to 30, with higher scores signifying more serious PPD [19]. Compared to other questionnaires, it has a satisfactory diagnostic efficiency and is more concise to subjects [20]. The sensitivity of EPDS has been proven to range from 0.67 to 1.00, and the specificity is consistently 0.87 or higher when the cut-off value is 13 [21]. Therefore, a score of EPDS ≥ 13 was determined to be positive for PPD screening in our study.

The Leakage Index Questionnaire (involving 3 items with multiple choices) and pain scale were used to evaluate the recovery of muscles in the pelvic floor and the degree of postpartum pain in mothers, respectively. The scores of the former range from 0 to 6 and from 0 to 10 in the latter. The higher scores on the Leakage Index Questionnaire predict poorer recovery of pelvic floor muscles. Moreover, educational background, annual family income, and milk feeding methods were also investigated in our questionnaire (details are shown in Figure S1).

Besides, severe PE was diagnosed as (1) systolic pressure ≥ 160 mmHg or diastolic pressure ≥ 110 mmHg, measured at least every 4 hours; (2) platelet count ≤ 100∗10^9^/L; (3) abnormal liver function (elevated liver enzymes twice the upper limit) without other diseases; (4) renal dysfunction (Scr ≥ 97.24 *μ*mol/L) in the absence of other diseases; (5) pneumonedema; (6) new-onset headache without other diseases; and (7) blurred vision [22]. FGR was defined as an infant birthweight below the 10^th^ percentile of the average of infants at the same gestational age. Patients without any complications during the perinatal period were eligible for enrolment in the normal group.

### 2.4. Statistical Analysis

This study was designed to detect a 15% absolute difference between groups with 90% power and a 5% type I error rate. We assume that the incidence of PPD was about 30% in the PE group and 15% in the control group. Therefore, a sample size of 380 (88 in the PE group and 292 in the control group) was needed. The MedSci Sample Size Tools (MSST, version 5.7.15, copyright 2020 MedSci.cn) were applied for calculating. We recruited 130 PE patients and 295 healthy women.

Variables following normal distribution were compared via an independent *t*-test and presented as the mean ± standard deviation. Otherwise, variables were described as the mean ± quartile and examined by the Kruskal-Wallis test. Differences in the classified variables were evaluated by the chi-squared test. *P* < 0.05 was considered significant. A multivariate logistic regression model was used to evaluate adjusted odds ratios. Confounding factors include age, BMI, gestational days, baby weight, delivery model, Leakage Index Score, milk-feeding ways, and pain scale, which were previously reported to be connected with PPD or unmatched between PE and normal groups. All statistical analyses were conducted on SPSS 23.0 (SPSS Inc, Chicago, USA).

## 3. Results

### 3.1. Clinical Characteristics between Normal and PE Mothers

A total of 130 PE patients met the inclusion and exclusion criteria. We randomly selected 295 normal women who met the inclusion criteria during the same period. In the PE group, 74 patients were diagnosed with mild PE, the others with severe PE. Clinical characteristics were compared between the normal and PE groups in Table 1. The layer distribution of prepregnancy body mass index (BMI) was significantly different, and PE mothers had a higher BMI than the normal ones (*P* = 0.005). PE patients suffered a much higher rate of cesarean section (93.08% *vs.* 33.22%) and less gestational age (260 *vs.* 277 days) (both *P* < 0.001). As a result, the birthweight of the fetuses in the PE group was inferior to the normal one (2960 *vs.* 3255 g, *P* < 0.001). In terms of feeding, infant formula was more frequently used in the PE group, no wonder the exclusive breastfeeding rate was lower (33.08% *vs.* 57.63%, *P* < 0.001). However, other clinical characteristics were not significantly different between groups, such as BMI increase, gravidity, parity, percentage of primiparas, sex of the fetus, educational background, annual family income, and scores of the Leakage Index Questionnaire and pain scale.

### 3.2. Clinical Characteristics between PPD and Non-PPD Groups

All participants were asked to finish EPDS, and the scores were compared between the normal and PE groups. No differences in clinical characteristics were found between PPD and non-PPD mothers in the normal group (Table 2). However, in the PE group, mothers who had babies with FGR and low neonatal weight tended to develop PPD (*P* = 0.024 and *P* = 0.007; Table 3). Postpartum pain was another high-risk factor for PPD in the PE group (*P* = 0.012). Unlike in the PE group, the scores of the pain scale showed no difference between PPD and non-PPD women in the normal group (*P* = 0.209).

### 3.3. Severe PE and FGR Women Were Inclined to Develop Higher EPDS Scores

**Figure 1:**
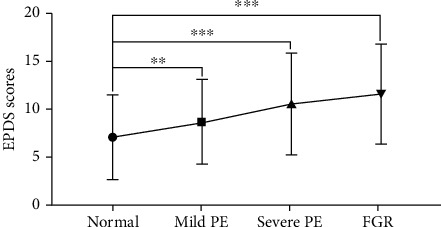
The average EPDS score in the normal group was significantly lower than that of the mild PE subgroup. The *P* values were shown as ^∗∗^*P* < 0.01; ^∗∗∗^*P* < 0.001.

We tried to explore the associations between PPD and PE. The average EPDS score in the normal group was significantly lower than that of the mild PE subgroup (7.09 ± 4.41 vs. 8.62 ± 4.35, *P* = 0.008; Table 4 and Figure 1). In the severe PE subgroup, the average EPDS score was even worse (10.58 ± 5.41), indicating that most of the severe PE patients developed PPD. Furthermore, 38 PE mothers were complicated with FGR and got the highest EPDS scores among the subgroups (11.61 ± 5.29, *P* < 0.001).

### 3.4. Rather than Cesarean Section, PE Showed a Direct Tendency on PPD Development

There was a higher cesarean section rate among PE patients than normal women (93.08% *vs.* 33.22%). To determine the effect of the cesarean section on PPD development, we compared the EPDS scores and PPD incidence between C-section and vaginal delivery in the normal group. There was not any difference in the EPDS score (6.74 ± 4.42*vs.*7.27 ± 4.41, *P* = 0.337) and PPD incidence (13.27% *vs.* 15.32%, *P* = 0.728) between the two delivery modes (Table 5). Interestingly, when comparing the normal and PE groups suffering from cesarean section, it was found that both the EPDS (9.54 ± 4.80*vs.*6.74 ± 4.42, *P* < 0.001) score and PPD incidence (32.23% *vs.* 13.27%, *P* = 0.001) were much higher among the PE group than the normal group (Table 6). It could be inferred that it was not cesarean section but PE that directly increased the risk of PPD.

### 3.5. Much Higher Screening of PPD in PE Mothers than the Normal Ones

We compared the rate of positive screening of PPD in each subgroup (Table 7). Totally, 83 people were screened positive for PPD, while the remaining 342 were negative. The incidence of PPD was 14.58% in the normal group, whereas the rate was much higher among PE mothers. About 30.77% of women in the PE group met the diagnostic criterion for PPD. Furthermore, the incidences of PPD dramatically increased with the severity of PE and its complications. For instance, the incidences of PPD in the mild PE and severe PE subgroups were 27.03% and 36.96%, respectively, which were significantly higher than that of the normal mothers (14.58%, *P* = 0.014 and *P* = 0.002).

We also tried to explore the associations between PE complications and PPD development. In the PE+FGR subgroup, the incidence of PPD was the highest among all the subgroups (44.74%). Thirty newborns were extremely weak and had to be sent to the neonatal intensive care unit (NICU). Obviously, when the babies were sent to NICU, their mothers tended to develop PPD. PPD incidence among these mothers increased dramatically (36.66%), which was extremely high. Preterm, one of the common complications in PE, occurred in almost half of PE mothers (61 of 130). PPD occurrence was 32.79% in the PE+preterm subgroup.

### 3.6. Independent Risk Factors for PPD

**Figure 2:**
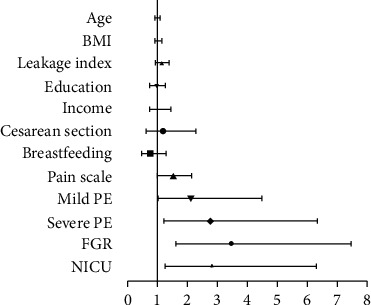
The odds ratio of PPD for each characteristic. The line segment represents the odds ratio and 95% confidence interval for each variable. It shows that the pain scale, mild PE, severe PE, FGR, and NICU were risk factors with OR and 95%CI > 1.

Then, multiple logistic regression was performed to evaluate the independent risk factors for PPD. With PPD as the dependent variable, PE, severe PE, FGR, and NICU admission were regarded as independent variables individually, while age, BMI, gestational days, baby weight, delivery model, Leakage Index Score, milk-feeding ways, and pain scale were analyzed as confounding factors. Women with mild PE demonstrated 2-fold higher odds of PPD (AOR = 2.117, 95% CI: 1.001-4.479; Table 8). Furthermore, severe PE, FGR, and NICU admission all increased nearly 3-fold risk for PPD positive screening. These findings indicate that the severity and complications of PE will increase the risk of PPD (as shown in Figure 2).

Besides PE, postpartum pain was another independent risk factor for PPD (AOR = 1.509, 95% CI: 1.078-2.114). The effect of breastfeeding on PPD has not been indicated before, but in our study, exclusive breastfeeding seemed not to positively affect the mood of the mothers (AOR = 0.752, 95% CI: 0.445-1.270). Pelvic floor muscle recovery has always been a concern among new mothers and can dramatically influence their moods. After evaluating pelvic floor function, we found that the dysfunction of pelvic floor muscles had no negative effect on PPD (AOR = 1.137, 95% CI: 0.952-1.358). Moreover, we observed that there were no correlations between the cesarean section and PPD (AOR = 1.177, 95% CI: 0.620-2.232).

## 4. Discussion

Among the general population, hypertension has already been proved to be an independent risk factor for depressive disorder [23]. Hypertension increased 1.12-fold of developing depression among 6,237 old Chinese adults [24]. For pregnant women, few studies were exploring the connection between PE and PPD. To our knowledge, this is the first retrospective cohort study to clarify the associations of the severity and complications of PE with PPD in the Chinese population. The number of cases (425) in our trial is the largest among the existing relevant studies.

As reported, PPD occurred in 20.5% of PE patients in Tanzanian and in about 21% of PE mothers in Greek [11, 25]. In our study, the percentage of positive screening for PPD in the PE group was even higher (30.77%). Besides PE, its complications could also increase the risk of PPD. Similar to our findings, Hoedjes et al. discovered that the prevalence of PPD was 23% in mild PE patients and 44% in severe PE [26]. These studies suggest that PE affects PPD strongly.

For PE mothers, besides the unfavorable experience of hypertension, other conditions such as additional costs and concerns of the newborns with complications also increase mothers' psychological burden [27, 28]. The outcomes of infants play an important role in PPD development among severe PE patients [26]. This conclusion was confirmed in our study, especially for growth-restricted babies. A study reported that the prevalence of PPD among the FGR family was 48.2%, which was similar to our result (44.74%) [27]. 38% of mothers experienced significant depressive symptoms when their babies were sent to NICU [29]. These studies mentioned that baby conditions and financial problems may be two of the most risk factors for PPD. In our clinical trial, mothers were asked, “What most upsets you?” The majority of mothers told us that they were bothered most by the poor outcomes of their babies and NICU admission. In our study, 30 neonates were admitted to NICU. Notably, the incidence of PPD among these mothers was very high (36.6%). Many randomized controlled trials have implicated that insufficient contact with babies will increase the odds of PPD [30–32]. Therefore, clinical healthcare workers should provide psychological supplies to mothers with NICU babies. The financial problem was the second problem: seven of them received lower annual income (less than 80 k RMB per year); they felt huge burdens on children's hospital expenses.

Whether the cesarean section will increase the risk of PPD is still controversial. In China, some healthy pregnant women would like to choose a cesarean section due to social-psychological factors. In this research, mothers with PE preferred to have a cesarean section to avoid possible adverse outcomes. This can explain why the rate of operative delivery in China among PE patients is so high. First of all, to figure out the effect of the cesarean section for PPD, we compared delivery models among normal women. Patel et al. demonstrated that operative delivery would not increase the incidence of PPD in 14,633 women [33]. A meta-analysis in 2017 also reported that elective cesarean section would not significantly exacerbate the odds of PPD (AOR: 1.15, 95% CI: 0.92-1.43) [34]. In our study, there was not any difference in the EPDS score and PPD incidence between the two delivery models among normal pregnant populations.

However, in the PE group, we found that both EPDS score and PPD incidence were much higher in mothers suffering from the operation. It could be inferred that PE directly increased the risk of PPD rather than cesarean section. Then, we applied subgroup analysis to find the reason. In the PE+FGR subgroup, the incidence of PPD was the highest among all the subgroups. Obviously, mothers tended to show anxiety when babies were sent to NICU. Another common complication is preterm. Almost half of PE mothers occurred preterm. As expected, mothers in the PE+preterm group experienced higher psychological distress than others. Weigl et al. pointed out that new mothers of preterm infants exhibited higher scores of depression, anxiety, and stress than parents of term infants [35]. Preterm mothers showed lower levels of estradiol, progesterone, and prolactin, as well as a heightened postawakening cortisol response, compared to term mothers. These results are consistent with our findings.

Postpartum pain, urinary incontinence, and feeding methods were also evaluated in the regression model. Postpartum pain was an independent risk factor for PPD, increasing the odds by 1.5-fold. A few trials showed that untreated pain is associated with a risk of PPD [36, 37]. The usage of painkillers can help decrease the incidence of PPD in some cases [38, 39]. Our study implied postpartum pain as another risk factor for PPD in the PE group. This was probably because PE mothers suffered more postpartum pain from the operation. Therefore, it is reasonable to use painkillers for PE mothers during the postnatal period.

Hullfish et al. have demonstrated a correlation between urinary incontinence and PPD [40], but Doering et al. showed no such connection [41]. In our study, there was no significant result about urinary incontinence in PPD development. Nonetheless, more authoritative urinary incontinence scales need to be tested in the future. Nonbreastfeeding was regarded as a risk factor for PPD in many cases [42, 43]. But in our study, exclusive breastfeeding did not help decrease the incidence of PPD.

Although the connection between PE and PPD is still unclear, some mechanisms, such as clinical symptoms, inflammation, and genetic changes, have been used as hypotheses for the reason between PE and PPD. The pathogenesis for PE, a placenta disease, can be explained by the “two-stage theory” [44]. At the first stage, vascular remodeling disorders of uterine spiral arterioles caused by multiple factors result in “superficial placental implantation” and ultimately cause insufficient placental perfusion and impairment of placental function. In the second stage, the ischemic placenta will experience oxidative stress and release inflammatory factors, such as IL-6, leading to systemic endothelial dysfunction. Therefore, PE patients often have excessive inflammatory factors in blood circulation [45–48]. For example, abnormally elevated C-reactive protein (CRP) and tumor necrosis factor- (TNF-) *α* are detected in the serum of PE mothers, resulting in vascular remodeling dysfunction of the placenta [49]. Consistently, like PE, inflammatory biomarkers also take part in PPD development [50]. Studies confirm that increased IL-6 and TNF-*α* levels during the perinatal period can intensify the risk of PPD [51–53]. Based on these studies, we speculate that inflammatory cytokines are released by the dysfunctional placenta in PE mothers, finally leading to the development of PPD [48, 53, 54]. Our further research will pay more attention to these inflammatory cytokines.

## 5. Limitations

We must admit that there are some limitations in our study. As a retrospective study, it suffered from bias and case limitations. Firstly, patients were recruited from 2 hospitals, and the local bias may be relatively reduced, but there is still a need for a study involving multiple centers. Secondly, it was hard to control the operation rate in the PE group, although this delivery mode was not found to be a risk factor in our regression model. Thirdly, EPDS was a preliminary screening tool, not the gold standard for the diagnosis of PPD. In the future, we would like to initiate larger randomized controlled trials and more in-depth mechanistic studies.

## 6. Conclusions

PE can be an independent risk factor for PPD. Moreover, its severity and complications exacerbate the development of PPD. Severe PE, FGR, and NICU admission all increased nearly 3-fold risk for PPD-positive screening. Patients with PE should be offered suitable interventions, such as pain management, more cognitive-behavioral therapies (CBT), and interpersonal psychotherapies (IPT) to prevent the development of PPD.

## Figures and Tables

**Table 1:** Comparison of baseline characteristics between normal and PE women.

Variables	PE (*N* = 130) (%)	Normal (*N* = 295) (%)	*P* value
Age (y)			0.135^a^
18-24	9 (6.92)	24 (8.13)	
25-34	104 (80.0)	250 (84.75)	
35-45	17 (13.08)	21 (7.12)	
Prepregnant BMI (kg/m^2^)			0.005^a^
<18.5	20 (15.4)	59 (20.0)	
18.5-23.9	86 (66.2)	215 (72.9)	
24.0-27.9	21 (16.1)	17 (5.7)	
≥28.0	3 (2.3)	4 (1.4)	
BMI increment	5.71 ± 1.74	5.76 ± 1.61	0.792^b^
Gravidity	2 (1-3)	2 (1-3)	0.942^c^
Parity	1 (1-2)	1 (1-2)	0.777^c^
Primipara	91 (70.0)	213 (72.20)	0.643^a^
Cesarean section	121 (93.08)	98 (33.22)	<0.001^a^
Gestational weeks (d)	260 (251.75-269.00)	277 (272.00-282.00)	<0.001^c^
Male baby	65 (50.0)	167 (56.61)	0.245^a^
Neonatal weight (g)	2960 (2197.50-3362.50)	3255 (3035.00-3560.00)	<0.001^c^
Education background^∗^	4 (3-4)	4 (3-4)	0.451^c^
Annual income^∗∗^	3 (2-3)	3 (2-3)	0.839^c^
Leakage Index	0 (0-1)	0 (0-2)	0.713^c^
Pain scale	1 (1-1)	1 (1-1)	0.268^c^
Exclusive breastfeeding	43 (33.08)	170 (57.63)	<0.001^a^

^a^The *P* value is calculated by the chi-squared test. ^b^The *P* value is calculated by the independent sample *t*-test. ^c^The *P* value is calculated by the Kruskal-Wallis test. Education background^∗^ was divided into 5 degrees: 1: no more than junior middle school; 2: senior high school; 3: junior college; 4: bachelor degree; 5: master degree or above. Annual income^∗∗^ (of family) was divided into 5 degrees: 1: 30k-80k RMB per year; 2: 80k-120k RMB per year; 3: 120k-200k RMB per year; 4: 200k-300k RMB per year; 5: more than 300k RMB per year.

**Table 2:** Comparison of baseline characteristics between PPD and non-PPD women in the normal group.

Variables	PPD group (*N* = 43)	Non-PPD group (*N* = 252)	*P* value
Age	28.12 ± 3.26	29.03 ± 3.67	0.126^b^
Prepregnant BMI	20.35 ± 2.55	20.72 ± 2.41	0.387^b^
BMI increment	5.52 ± 1.57	5.79 ± 1.62	0.284^b^
Gravidity	2 (1-3)	2 (1-3)	0.450^c^
Parity	1 (1-2)	1 (1-2)	0.242^c^
Primipara	28 (65.12)	185 (73.41)	0.173^a^
Cesarean section	13 (30.23)	85 (33.73)	0.397^a^
Gestational weeks (d)	276 (273-283)	277 (272-282)	0.611^c^
Male baby	26 (60.47)	141 (55.95)	0.352^a^
Neonatal weight (g)	3240.00 (3040.00-3700.00)	3260.00 (3030.00-3530.00)	0.352^c^
Education background	3 (3-4)	4 (3-4)	0.290^c^
Annual income	3 (2-3)	3 (2-3)	0.851^c^
Leakage Index Score	1 (0-2)	0 (0-2)	0.419^c^
Pain scale	1 (1-2)	1 (1-1)	0.209^c^
Exclusive breastfeeding	21 (48.84)	149 (59.13)	0.137^a^

^a^The *P* value is calculated by the chi-squared test. ^b^The *P* value is calculated by an independent sample *t*-test. ^c^The *P* value is calculated by the Kruskal-Wallis test.

**Table 3:** Comparison of baseline characteristics between PPD and Non-PPD women in the PE group.

Variables	PPD group (*N* = 40) (%)	Non-PPD group (*N* = 90) (%)	*P* value
Severe PE	16 (40.0)	30 (33.3)	0.295^a^
FGR	17 (42.5)	21 (23.3)	0.024^a^
Age	30.08 ± 4.33	29.86 ± 3.84	0.783^b^
Prepregnant BMI	21.48 ± 2.97	21.24 ± 2.85	0.684^b^
BMI increment	5.91 ± 1.90	5.61 ± 1.67	0.396^b^
Gravidity	2 (1-3)	2 (1-3)	0.514^c^
Parity	1 (1-2)	1 (1-2)	1.000^c^
Primipara	28 (70.0)	63 (70.0)	0.578^a^
Cesarean section	39 (97.5)	82 (91.1)	0.173^a^
Gestational weeks (d)	259.00 (247.25-265.75)	261.00 (252.75-269.00)	0.368^c^
Male baby	21 (52.5)	44 (48.9)	0.425^a^
Neonatal weight (g)	2415.00 (1822.50-3187.50)	3065.00 (2352.50-3442.50)	0.007^c^
Education background	4 (3-4)	4 (3-4)	0.891^c^
Annual income	3 (2-3)	3 (2-3)	0.651^c^
Leakage Index Score	1 (0-2)	0 (0-1)	0.208^c^
Pain scale	1 (1-2)	1 (1-1)	0.012^c^
Exclusive breastfeeding	11 (27.5)	32 (35.5)	0.244^a^
NICU	11 (27.5)	19 (21.11)	0.280^a^

^a^The *P* value is calculated by the chi-squared test. ^b^The *P* value is calculated by an independent sample *t*-test. ^c^The *P* value is calculated by the Kruskal-Wallis test.

**Table 4:** Comparison of EPDS scores in each subgroup.

Variables	Samples (*N*)	EPDS scores	*t*-test	*P* value^a^
Normal	295	7.09 ± 4.41	Reference	Reference
Mild PE	74	8.62 ± 4.35	-2.690	0.008
Severe PE	46	10.58 ± 5.41	-4.170	<0.001
PE+FGR	38	11.61 ± 5.29	-5.031	<0.001

^a^All of the *P* values are calculated by an independent sample *t*-test.

**Table 5:** Comparison of characteristics between two different delivery modes in normal women.

Variables	C-sections (*N* = 98) (%)	Vaginal delivery (*N* = 197) (%)	*P* value
Age	29.65 ± 3.88	28.52 ± 3.43	0.011^b^
Prepregnant BMI	20.91 ± 2.47	20.54 ± 2.41	0.239^b^
BMI increment	5.87 ± 1.66	5.70 ± 1.59	0.381^b^
Gravidity	2 (1-3)	2 (1-2)	0.257^c^
Parity	1 (1-2)	1 (1-2)	0.270^c^
Primipara	67 (68.37)	146 (74.11)	0.335^a^
Gestational weeks (d)	276 (272-282)	277 (272-282)	0.635^c^
Male baby	54 (55.10)	113 (57.36)	0.803^a^
Neonatal weight (g)	3252.5 (3050.0-3600.0)	3260.0 (3020.0-3535.0)	0.275^c^
Education background	4 (3-4)	4 (3-4)	0.120^c^
Annual income	2 (2-3)	3 (2-3)	0.024^c^
Leakage Index	0 (0-1)	1 (0-2)	<0.001^c^
Pain scale	1 (1-1)	1 (1-1)	0.025^c^
Exclusive breastfeeding	54 (55.10)	116 (58.88)	0.617^a^
EPDS score	6.74 ± 4.42	7.27 ± 4.41	0.337^b^
PPD	13 (13.27)	30 (15.23)	0.728^a^

^a^The *P* value is calculated by a chi-squared test. ^b^The *P* value is calculated by an independent sample *t*-test. ^c^The *P* value is calculated by the Kruskal-Wallis test.

**Table 6:** Comparison of baseline characteristics in normal and PE women who suffered cesarean section.

Variables	PE (*N* = 121) (%)	Normal (*N* = 98) (%)	*P* value
Age	30.02 ± 3.81	29.65 ± 3.88	0.488^b^
Prepregnant BMI	21.27 ± 2.87	20.91 ± 2.47	0.316^b^
BMI increment	5.74 ± 1.76	5.87 ± 1.66	0.576^b^
Gravidity	2 (1-3)	2 (1-3)	0.240^c^
Parity	1 (1-2)	1 (1-2)	0.623^c^
Primipara	85 (70.25)	67 (68.37)	0.770^a^
Gestational weeks (d)	260 (251-269)	276 (272-282)	<0.001^c^
Male baby	60 (49.59)	54 (55.10)	0.497^a^
Neonatal weight (g)	2960.0 (2170.0-3400.0)	3252.5 (3050.0-3600.0)	<0.001^c^
Education background	4 (3-4)	4 (3-4)	0.141^c^
Annual income	3 (2-3)	2 (2-3)	0.172^c^
Leakage Index	0 (0-1)	0 (0-1)	0.073^c^
Pain scale	1 (1-1)	1 (1-1)	0.240^c^
Exclusive breastfeeding	40 (33.06)	54 (55.10)	0.002^a^
EPDS score	9.54 ± 4.80	6.74 ± 4.42	<0.001^b^
PPD	39 (32.23)	13 (13.27)	0.001^a^

^a^The *P* value is calculated by the chi-squared test. ^b^The *P* value is calculated by an independent sample *t*-test. ^c^The *P* value is calculated by the Kruskal-Wallis test.

**Table 7:** Comparison of the incidence of PPD between normal and PE women.

Variables	PPD (%)	Non-PPD (%)	*P* value^a^
Normal (*N* = 295)	43 (14.58)	252 (85.42)	Reference
Total PE (*N* = 130)	40 (30.77)	90 (69.23)	<0.001
Mild PE (*N* = 74)^#^	20 (27.03)	54 (72.97)	0.014
Severe PE (*N* = 46)	16 (36.96)	30 (63.04)	0.002
PE+FGR (*N* = 38)	17 (44.74)	21 (52.26)	<0.001
NICU (*N* = 30)	11 (36.66)	19 (63.33)	0.004
PE+preterm (*N* = 61)	20 (32.79)	41 (67.21)	0.001

^a^All of the *P* values are calculated by the chi-squared test. ^#^In this group, 10 subjects with mild PE but with FGR were excluded.

**Table 8:** Multivariable logistic regression analysis for PPD in PE and normal patients.

Variables	OR (95% CI)	Adjusted OR (95% CI)^a^
Age	0.987 (0.925-1.052)	
Prepregnant BMI	1.006 (0.917-1.103)	
Gestational weeks (d)	0.985 (0.851-1.139)	
Baby weight	0.999 (0.999-1.000)	
Leakage Index	1.137 (0.952-1.358)	
Educational background	0.953 (0.731-1.241)	
Annual income	1.020 (0.741-1.403)	
Cesarean section	1.758 (1.074-2.887)	1.177 (0.620-2.232)^#^
Exclusive breastfeeding	0.558 (0.342-0.911)	0.752 (0.445-1.270)^##^
Pain scale	1.581 (1.151-2.174)	1.509 (1.078-2.114)^∗^
Mild PE	2.171 (1.184-3.981)	2.117 (1.001-4.479)^∗^
Severe PE	3.126 (1.527-6.216)	2.759 (1.206-6.315)^∗^
FGR	4.744 (2.317-9.713)	3.450 (1.596-7.458)^∗^
NICU	2.597 (1.184-5.696)	2.809 (1.258-6.270)^∗^

^a^The adjusted ORs were calculated by multifactor logistic regression models. ^#^Adjusted factors: age, BMI, and PE. ^##^Adjusted factors: age, BMI, PE, and cesarean section. ^∗^Adjusted factors: age, BMI, cesarean section, uroclepsia, breastfeeding, educational background, and annual income.

## Data Availability

The data used and analyzed during the current study are available from the corresponding authors on reasonable request.

## Supplementary Materials

Supplementary Materials Figure S1: the questionnaires were used in the study, including EPDS, Leakage Index Questionnaire, and pain scale.
