# Supplementary figures and images for: Preeclampsia and Its Complications Exacerbate Development of Postpartum Depression: A Retrospective Cohort Study
Source: Biomed Res Int. 2021 Apr 22;2021:6641510. doi: 10.1155/2021/6641510 (PMC8087462; doi:10.1155/2021/6641510)

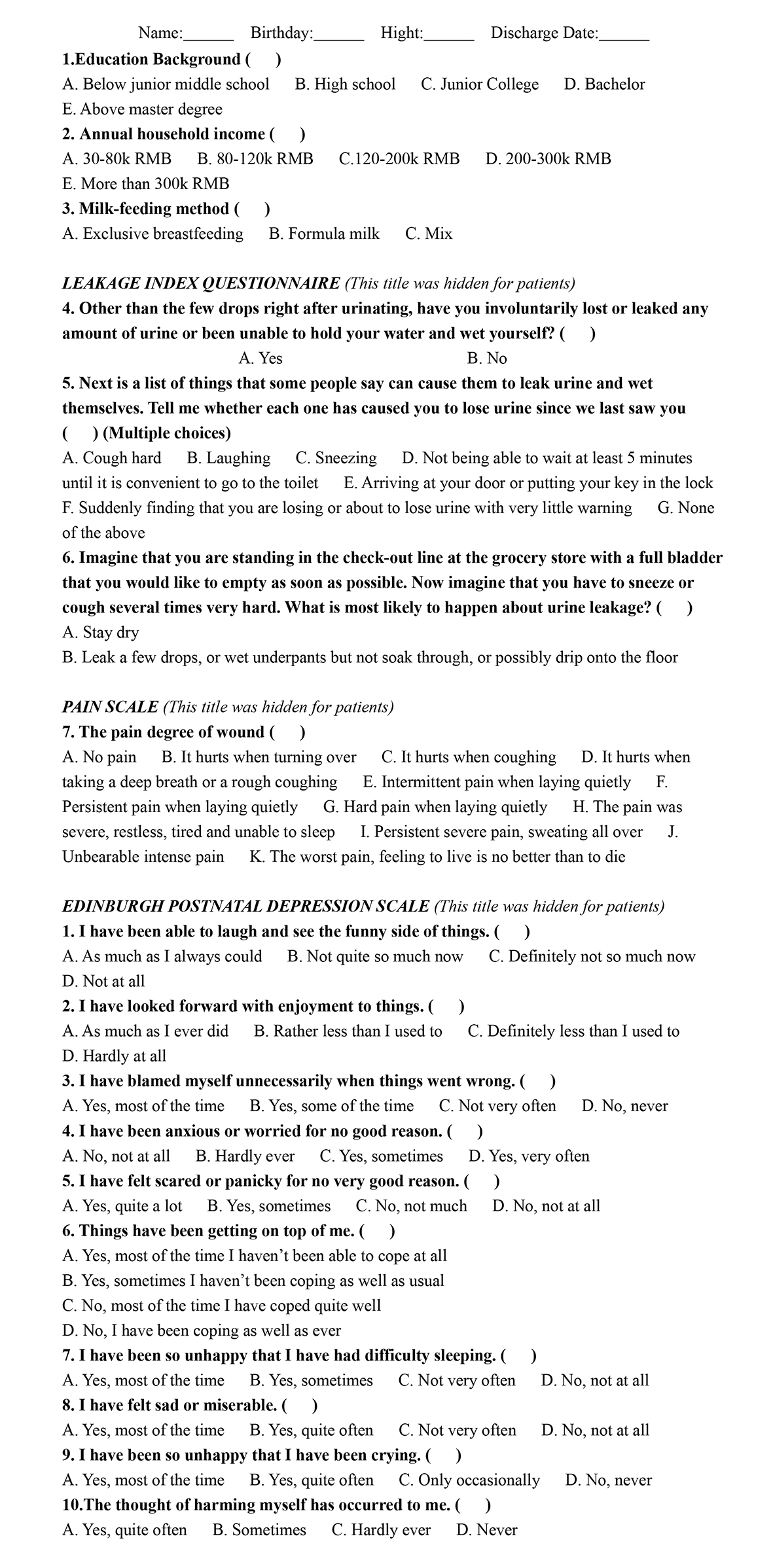

Supplement: Supplementary Materials — Figure S1: the questionnaires were used in the study, including EPDS, Leakage Index Questionnaire, and pain scale. [file 6641510.f1.jpg]
